# Prevalence and geographic variation of abdominal obesity in 7- and 9-year-old children in Greece; World Health Organization Childhood Obesity Surveillance Initiative 2010

**DOI:** 10.1186/s12889-017-4061-x

**Published:** 2017-01-28

**Authors:** Maria Hassapidou, Themistoklis Tzotzas, Evangelia Makri, Ioannis Pagkalos, Ioannis Kaklamanos, Efthymios Kapantais, Annet Abrahamian, Antonis Polymeris, Konstantinos Tziomalos

**Affiliations:** 1 Department of Nutrition and Dietetics, Alexander Technological Educational Institute of Thessaloniki, Thessaloniki, Greece; 2 First Propedeutic Department of Internal Medicine, Medical School, Aristotle University of Thessaloniki, AHEPA Hospital, 1 Stilponos Kyriakidi street, Thessaloniki, 54636 Greece; 3 Hellenic Medical Association for Obesity, Athens, Greece

**Keywords:** Obesity, Abdominal obesity, Children, Cardiovascular risk, Residence, Rural, Urban, Gender

## Abstract

**Background:**

In children, abdominal obesity is a better predictor of the presence of cardiovascular risk factors than body mass index (BMI)-defined obesity. We aimed to evaluate the prevalence of abdominal obesity in the Greek pediatric population and to assess the impact of residence on the prevalence of both BMI-defined and abdominal obesity.

**Methods:**

In the context of the Childhood Obesity Surveillance Initiative of the World Health Organization (WHO) Regional Office for Europe, a national representative sample of 7.0–7.9 and 9.0–9.9-year-old children was evaluated (*n* = 2,531 and 2,700, respectively). Overweight and obesity according to BMI were estimated using both the WHO and International Obesity Task Force cut-off points. Abdominal obesity was defined as waist circumference/height ratio >0.5.

**Results:**

The prevalence of abdominal obesity did not differ between 7-year-old boys and girls (25.2 and 25.3%, respectively; *p* = NS). Among 9-year-old children, abdominal obesity was more prevalent in boys than in girls (33.2 and 28.2%, respectively; *p* = 0.005). Among normal weight and overweight children, the prevalence of abdominal obesity was 1.6–6.8 and 21.8–49.1%, respectively. The prevalence of abdominal and BMI-defined obesity did not differ between children living in the mainland, in Crete and in other islands except in 7-year-old girls, where the prevalence of BMI-defined obesity was highest in those living in Crete, intermediate in those living in other islands and lowest in those living in the mainland. In 9-year-old boys and in 7- and 9-year-old girls, the prevalence of abdominal obesity was highest in children living in Athens and lowest in children living in Thessaloniki, whereas children living in other cities and in villages showed intermediate rates. The prevalence of abdominal obesity in 7-year-old boys and the prevalence of BMI-defined obesity did not differ between children living in cities and villages.

**Conclusions:**

The prevalence of pediatric abdominal obesity in Greece is among the highest worldwide. Boys and children living in the capital are at higher risk for becoming obese. Given that abdominal obesity is more prevalent than BMI-defined obesity and appears to be more sensitive in identifying cardiovascular risk, measurement of waist circumference might have to be incorporated in the screening for childhood obesity.

**Electronic supplementary material:**

The online version of this article (doi:10.1186/s12889-017-4061-x) contains supplementary material, which is available to authorized users.

## Background

Childhood obesity is a major public health problem [1]. The prevalence of childhood obesity has increased substantially in the last decades worldwide [2]. In 2013, almost one fourth of children in developed countries and more than 10% of children in developing countries were overweight or obese based on body mass index (BMI) [2]. Obese children are at increased risk for becoming obese adults [3–7] and also have elevated risk for developing hypertension, type 2 diabetes mellitus and cardiovascular disease [4, 8–11].

In children, abdominal obesity is a better predictor of the presence of cardiovascular risk factors than obesity evaluated with the BMI [12–15]. Moreover, a substantial proportion of children with normal BMI has abdominal obesity [16–19] and these children appear to have more adverse metabolic profile than children who are overweight/obese according to BMI but do not have abdominal obesity [15, 17]. Furthermore, the prevalence of abdominal obesity appears to have increased in the recent decades more steeply than the prevalence of obesity defined according to BMI [18, 20–22]. It has also been reported that rates of abdominal obesity and of obesity defined according to the BMI differ according to residence. However, different studies reported conflicting data on whether living in rural or in urban areas is associated with increased risk for obesity [23–28]. There are also limited data on whether rates of obesity differ between children living in the mainland and in those living in islands of the same country [16, 24, 29].

The World Health Organization (WHO) Regional Office for Europe has established the Childhood Obesity Surveillance Initiative (COSI) to monitor the trends of obesity in primary-school children [30]. In the context of this program, it was recently reported that the prevalence of obesity in children 7 or 9 years old in Greece is the highest in Europe [30]. The aim of the present study was to evaluate the prevalence of abdominal obesity in the same Greek pediatric population and to assess the impact of the residence on the prevalence of both BMI-defined and abdominal obesity.

## Methods

Methods have been described in detail before [30]. Briefly, COSI Round 2 was performed in a national representative sample of schools in Greece between November 2010 and March 2011. Two age groups were evaluated: 7.0–7.9 and 9.0–9.9 years old. Since children of these age groups in Greece are enrolled in primary schools, the school population was considered to be representative of the total population in these age groups. Cluster sampling was employed whereby the primary sampling unit was the primary school or the class. Primary schools and classes were selected randomly from the list of all primary schools obtained from the Ministry of Education. In every sampled class, all children were invited to participate. Primary sampling units were stratified by prefecture.

A total of 7,432 students were registered in the selected classes. 1,464 parents did not return the written consent and 267 children were absent on the day of the measurements. No child refused to be measured. Finally, 5,701 children were evaluated (response rate, 76.7%). Among these children, 2,531 were 7.0–7.9 years old and 2,700 were 9.0–9.9 years old. These 5,231 children constituted the study population.

Prior to data collection, all examiners (experienced clinical dietitians) were trained in measuring weight and height using WHO standardized techniques [31]. Children were asked to take off their shoes and socks as well as all heavy clothing (coats, sweaters, jackets, etc.) and to remove items such as wallets, mobile phones or key chains. Body weight was measured to the nearest 0.1 kg with portable digital scales (Tanita UM 075, Amsterdam, The Netherlands) and body height was measured standing upright to the nearest 0.1 cm with portable stadiometers (Tanita HR 001, Amsterdam, The Netherlands). Body weight was then adjusted for the weight of the clothes worn. The waist circumference (W) was measured at midway between the lowest border of the rib cage and the iliac crest.

Overweight and obesity according to BMI were estimated using both the WHO and International Obesity Task Force (IOTF) BMI cut-off points (Additional File 1) [32, 33]. We applied both cut-offs because they yield different estimates of the prevalence of overweight and obesity but it is unclear which provides the more accurate estimate [34–36]. Abdominal obesity was defined as W/height ratio >0.5 [13].

Based on their residence, children were divided into: a) those living in the mainland, in Crete and in other islands and b) those living in Athens (the capital of Greece, with a population of approximately 3.7 million people), in Thessaloniki (the second largest city in Greece, with a population of approximately 1 million people), in smaller cities (10,000–150,000 residents) and in villages (<10,000 residents).

The COSI protocol was in accordance with the international Ethical Guidelines for Biomedical Research Involving Human Subjects [37]. The procedures were also approved by the Ethics Committee of the Alexander Technological Educational Institute of Thessaloniki. Parents’ and children’s consent was obtained prior to the measurements.

### Statistical analysis

All data were analyzed with the statistical package SPSS (version 17.0; SPSS, Chicago, IL, USA). Data are presented as percentages for categorical variables and as mean and standard deviation for continuous variables. Differences in categorical variables between groups were assessed with the chi-square test. Differences in continuous variables between groups were assessed with one-way analysis of variance and pair-wise post-hoc comparisons between groups were performed with the Holm-Sidak test. In all cases, a two-tailed *p* <0.05 was considered significant.

## Results

The prevalence of obesity in the total study population according to age and gender is shown in Table 1. Among 7-year-old children, the prevalence of abdominal obesity did not differ between boys and girls (25.2 and 25.3%, respectively; *p* = NS). In contrast, among 9-year-old children, abdominal obesity was more prevalent in boys than in girls (33.2 and 28.2%, respectively; *p* = 0.005). On the other hand, the prevalence of obesity defined according to BMI was higher in boys in both age groups when the WHO definition was applied but did not differ between genders in either age group when the IOTF definition was used (Table 1).

**Table 1:** Prevalence of obesity according to age and gender

	Boys 7 years old (*n* = 1280)	Girls 7 years old (*n* = 1251)	*p*
BMI (kg/m^2^)	17.9 ± 2.8*	17.8 ± 2.8*	0.463
W (cm)	60.8 ± 8.1*	60.1 ± 8.1*	0.033
Overweight/obese (%)(IOTF)	25.4/14.7**	25.9/14.8	0.952
Overweight or obese (%)(IOTF)	40.1**	40.7	0.791
Overweight/obese (%)(WHO)	24.4/25.8*	26.5/19.7	0.001
Overweight or obese (%)(WHO)	50.2*	46.2***	0.047
Abdominal obesity (%)	25.2*	25.3	0.952
	Boys 9 years old (*n* = 1276)	Girls 9 years old (*n* = 1424)	*p*
BMI (kg/m^2^)	19.6 ± 3.5*	19.3 ± 3.6*	0.028
W (cm)	67.1 ± 10.7*	65.3 ± 10.1*	<0.001
Overweight/obese (%)(IOTF)	31.0/14.9**	28.0/14.7	0.192
Overweight or obese (%)(IOTF)	45.9**	42.7	0.105
Overweight/obese (%)(WHO)	26.0/31.7*	29.2/21.5	<0.001
Overweight or obese (%)(WHO)	57.7*	50.7***	<0.001
Abdominal obesity (%)	33.3*	28.2	0.005

*BMI* body mass index, *W* waist circumference, *IOTF* overweight and obesity defined according to the International Obesity Task Force, *WHO* overweight and obesity defined according to the World Health Organization

Comparisons of BMI, W and of rates of obesity and abdominal obesity between 7– and 9-year-old children of the same gender: * *p* <0.001; ** *p* <0.005 *** *p* <0.05

Among boys, BMI, W and the prevalence of abdominal obesity and of obesity defined according to BMI were higher in those 9 years old than in those 7 years old (Table 1). In girls, BMI and W were also higher in those 9 years old than in those 7 years old (*p* <0.001 for both comparisons). However, the prevalence of abdominal obesity and of obesity defined according to BMI was similar in the two age groups except the prevalence of obesity according to the WHO definition of BMI cutoffs, which was higher in 9-year-old girls (*p* = 0.021)(Table 1).

The prevalence of abdominal obesity in the different BMI categories is shown in Table 2. Among normal weight and overweight children based on the IOTF definition, the prevalence of abdominal obesity was 3.4–6.8 and 38.4–49.1%, respectively. Among normal weight and overweight children based on the WHO definition, the prevalence of abdominal obesity was 1.6–3.3 and 21.8–30.5%, respectively.

**Table 2:** Prevalence of abdominal obesity according to body mass index categories

	International Obesity Task Force definition of obesity			World Health Organization definition of obesity		
	Normal weight	Overweight	Obese	Normal weight	Overweight	Obese
Boys 7 years-old	3.4	39.4	88.8	1.6	21.8	73.9
Girls 7 years-old	3.8	38.4	88.6	2.2	30.5	81.7
Boys 9 years-old	6.8	49.1	96.8	2.4	28.0	79.0
Girls 9 years-old	4.2	44.1	90.9	3.3	27.0	87.3

The prevalence of abdominal obesity differs significantly between categories of body mass index in both boys and girls, in both age groups and according to both definitions of obesity (*p* <0.001 for all comparisons)

The prevalence of obesity in children living in the mainland, Crete and in other islands is shown in Table 3. In both genders and in both age groups, the prevalence of abdominal obesity did not differ between children living in the mainland, in Crete and in other islands. Moreover, in 7-year-old boys and in girls of both age groups, the prevalence of obesity defined according to BMI also did not differ between children living in the mainland, in Crete and in other islands. In contrast, in 7-year-old girls, the prevalence of obesity defined according to BMI was highest in those living in Crete, intermediate in those living in other islands and lowest in those living in the mainland when either the WHO or IOTF definition was applied (*p* <0.05 for all comparisons; Table 3).

**Table 3:** Prevalence of obesity according to residence (mainland vs. Crete vs. other islands)

Boys 7 years-old				
	Mainland (*n* = 1097)	Crete (*n* = 126)	Other islands (*n* = 57)	*p*
BMI (kg/m^2^)	17.9 ± 2.9	17.9 ± 2.6	18.4 ± 3.1	0.439
W (cm)	60.7 ± 8.1	60.9 ± 7.9	62.4 ± 7.7	0.292
Overweight/obese (%)(IOTF)	25.5/14.0	22.2/19.8	29.8/17.5	0.333
Overweight or obese (%)(IOTF)	39.5	42.1	47.4	0.446
Overweight/obese (%)(WHO)	24.5/25.2	21.4/29.4	28.1/29.8	0.617
Overweight or obese (%)(WHO)	49.7	50.8	57.9	0.480
Abdominal obesity (%)	24.7	27.0	29.8	0.606
Girls 7 years-old				
	Mainland (*n* = 1077)	Crete (*n* = 118)	Other islands (*n* = 56)	*p*
BMI (kg/m^2^)	17.8 ± 2.8	18.3 ± 3.1	17.9 ± 2.8	0.182
W (cm)	60.1 ± 8.0	60.4 ± 8.3	59.7 ± 8.5	0.862
Overweight/obese (%)(IOTF)	24.7/14.6	35.9/15.4	26.8/17.9	0.085
Overweight or obese (%)(IOTF)	39.3	51.3	44.6	0.037
Overweight/obese (%)(WHO)	25.9/19.0	34.7/22.9	19.6/26.8	0.046
Overweight or obese (%)(WHO)	44.9	57.6	46.4	0.031
Abdominal obesity (%)	24.9	30.5	23.2	0.383
Boys 9 years-old				
	Mainland (*n* = 1089)	Crete (*n* = 140)	Other islands (*n* = 47)	*p*
BMI (kg/m^2^)	19.6 ± 3.6	19.5 ± 3.5	19.7 ± 3.5	0.918
W (cm)	67.1 ± 10.9	66.5 ± 9.6	67.7 ± 10.1	0.730
Overweight/obese (%)(IOTF)	30.6/15.1	32.9/13.6	34.0/14.9	0.960
Overweight or obese (%)(IOTF)	45.7	46.4	48.9	0.903
Overweight/obese (%)(WHO)	26.7/31.3	22.1/33.6	23.4/36.2	0.772
Overweight or obese (%)(WHO)	57.9	55.7	59.6	0.855
Abdominal obesity (%)	33.8	29.3	34.0	0.564
Girls 9 years-old				
	Mainland (*n* = 1235)	Crete (*n* = 141)	Other islands (*n* = 48)	*p*
BMI (kg/m^2^)	19.3 ± 3.6	19.2 ± 3.4	19.2 ± 3.9	0.914
W (cm)	65.3 ± 10.2	64.7 ± 9.3	66.4 ± 10.6	0.557
Overweight/obese (%)(IOTF)	28.5/14.6	28.6/14.3	14.6/18.8	0.341
Overweight or obese (%)(IOTF)	43.1	42.9	33.3	0.407
Overweight/obese (%)(WHO)	29.3/21.7	32.6/18.4	16.7/25.0	0.298
Overweight or obese (%)(WHO)	51.0	51.1	41.7	0.444
Abdominal obesity (%)	28.6	25.5	27.1	0.736

*BMI* body mass index, *W* waist circumference, *IOTF* overweight and obesity defined according to the International Obesity Task Force, *WHO* overweight and obesity defined according to the World Health Organization

The prevalence of obesity in children living in Athens, in Thessaloniki, in other cities and in villages is shown in Table 4. In 9-year-old boys and in 7- and 9-year-old girls, the prevalence of abdominal obesity was highest in children living in Athens and lowest in children living in Thessaloniki, whereas children living in other cities and in villages showed intermediate rates (*p* = 0.042, *p* = 0.035 and *p* = 0.001, respectively). In 7-year-old boys, the prevalence of abdominal obesity did not differ between children living in Athens, in Thessaloniki, in other cities and in villages. In addition, in both genders and in both age groups, the prevalence of obesity defined according to BMI did not differ between children living in Athens, in Thessaloniki, in other cities and in villages when either the WHO or IOTF definition was applied (Table 4).

**Table 4:** Prevalence of obesity according to residence (Athens vs. Thessaloniki vs. other cities vs. villages)

Boys 7 years-old					
	Athens (*n* = 222)	Thessaloniki (*n* = 211)	Other cities (*n* = 299)	Villages (*n* = 549)	*p*
BMI (kg/m^2^)	17.7 ± 2.8	17.8 ± 2.9	18.0 ± 2.9	17.9 ± 2.8	0.619
W (cm)	61.1 ± 8.0	60.1 ± 8.1	61.2 ± 8.2	60.7 ± 8.0	0.458
Overweight/obese (%)(IOTF)	24.3/13.1	21.4/15.2	23.5/15.8	28.4/14.6	0.427
Overweight or obese (%)(IOTF)	37.4	36.7	39.3	43.0	0.295
Overweight/obese (%)(WHO)	23.4/24.3	23.8/22.9	25.5/26.2	24.4/27.3	0.827
Overweight or obese (%)(WHO)	47.7	46.7	51.7	51.7	0.502
Abdominal obesity (%)	27.9	20.5	25.1	25.9	0.321
Girls 7 years-old					
	Athens (*n* = 237)	Thessaloniki (*n* = 239)	Other cities (*n* = 270)	Villages (*n* = 505)	*p*
BMI (kg/m^2^)	17.8 ± 2.9	17.7 ± 2.7	17.9 ± 2.7	17.9 ± 2.9	0.774
W (cm)	60.2 ± 8.7	58.9 ± 7.5	60.9 ± 8.1	60.2 ± 7.9	0.069
Overweight/obese (%)(IOTF)	21.6/15.7	26.4/13.8	27.1/15.2	27.0/14.5	0.794
Overweight or obese (%)(IOTF)	37.3	40.2	42.4	41.6	0.652
Overweight/obese (%)(WHO)	23.2/18.6	30.3/17.6	24.4/22.6	27.4/19.4	0.457
Overweight or obese (%)(WHO)	41.8	47.9	47.0	46.8	0.513
Abdominal obesity (%)	27.4	19.7	30.4	24.2	0.035
Boys 9 years-old					
	Athens (*n* = 187)	Thessaloniki (*n* = 325)	Other cities (*n* = 184)	Villages (*n* = 580)	*p*
BMI (kg/m^2^)	19.7 ± 3.9	19.4 ± 3.4	19.9 ± 3.7	19.7 ± 3.5	0.303
W (cm)	68.9 ± 11.4	65.5 ± 10.5	67.1 ± 11.8	67.4 ± 10.2	0.004
Overweight/obese (%)(IOTF)	27.3/16.0	32.6/12.3	31.1/16.4	31.3/15.5	0.723
Overweight or obese (%)(IOTF)	43.3	44.9	47.5	46.8	0.797
Overweight/obese (%)(WHO)	25.1/31.0	23.1/29.8	30.6/32.8	26.6/32.6	0.339
Overweight or obese (%)(WHO)	56.1	52.9	63.4	59.1	0.105
Abdominal obesity (%)	39.6	27.7	34.8	34.0	0.042
Girls 9 years-old					
	Athens (*n* = 240)	Thessaloniki (*n* = 346)	Other cities (*n* = 203)	Villages (*n* = 635)	*p*
BMI (kg/m^2^)	19.4 ± 3.7	19.2 ± 3.5	19.4 ± 3.6	19.3 ± 3.6	0.896
W (cm)	67.3 ± 10.6	63.8 ± 9.6	65.3 ± 9.8	65.3 ± 10.1	0.001
Overweight/obese (%)(IOTF)	31.1/14.7	31.3/11.0	27.6/16.3	25.2/16.3	0.161
Overweight or obese (%)(IOTF)	45.8	42.3	43.8	41.5	0.691
Overweight/obese (%)(WHO)	28.3/24.2	30.7/20.0	26.1/23.6	29.7/20.7	0.802
Overweight or obese (%)(WHO)	52.5	50.7	49.8	50.3	0.936
Abdominal obesity (%)	36.7	21.7	29.6	28.2	0.001

*BMI* body mass index, *W* waist circumference, *IOTF* overweight and obesity defined according to the International Obesity Task Force, *WHO* overweight and obesity defined according to the World Health Organization

Significant differences: Athens vs. Thessaloniki: *p* = 0.002

Significant differences: Athens vs. Thessaloniki: *p* <0.001

## Discussion

In the present study, performed in a nationally representative sample of 7- and 9-year old children living in Greece (*n* = 5,231), we observed a high prevalence of abdominal obesity (25–33%). Abdominal obesity was more frequent in 9-year-old boys than in girls of the same age but did not differ between genders in children 7 years old and was more prevalent in both boys and girls living in Athens than in those living in small cities and villages. On the other hand, the prevalence of obesity defined according to the BMI did not depend on residence, except in 7-year-old girls, in whom it was higher in those living in Crete than in those living in the mainland.

Approximately one fourth of Greek boys and girls 7 years old had abdominal obesity. In 9-year-old children, almost one third of boys and three out of ten girls had abdominal obesity. These rates are among the highest reported worldwide. Indeed, recent studies performed in children of similar age in Spain and Portugal reported a prevalence of abdominal obesity of 21.3 and 23.6%, respectively [16, 38]. In the National Health and Nutrition Examination Survey conducted in the United States between 2011 and 2012, 29.6% of children 6–11 years old had abdominal obesity [39]. In contrast, the prevalence of abdominal obesity was only 8.2 and 8.9% in children living in Sweden and Norway, respectively [25, 27]. In addition to these alarmingly high rates, it appears that the prevalence of abdominal obesity is increasing in Greece. Indeed, in a nationwide study conducted in 2003, the prevalence of abdominal obesity was 25.6 and 20.0% in boys and girls 6–12 years old, respectively [40]. In contrast, studies from other countries worldwide reported plateauing or decreasing rates of obesity in the last years [39, 41–43]. The high prevalence of abdominal obesity in Greece might be due to a number of factors, including the adoption of a sedentary lifestyle and a shift from the traditional Mediterranean diet to a western-type diet [44–46]. Indeed, previous studies reported increased intake of saturated fat and sweetened sugar beverages in the majority of Greek children [47–50].

Interestingly, 1.6–6.8 and 21.8–49.1% of normal weight and overweight children, respectively, had abdominal obesity in our study. These findings are in accordance with previous studies [16–19] and are of particular clinical importance, since normal weight children with abdominal obesity appear to have more adverse metabolic profile than overweight/obese children without abdominal obesity [15, 17]. Moreover, earlier studies reported that the prevalence of abdominal obesity appears to increase at a more rapid rate than the prevalence of obesity defined according to BMI [18, 20–22]. Therefore, assessment of abdominal obesity might have to be incorporated in the routine examination of children, in addition to the measurement of BMI. Nevertheless, current guidelines recommend using BMI for identifying obesity in children and do not mention indices of abdominal obesity as screening tools [51].

Among children 9 years old, the prevalence of abdominal obesity was higher in boys whereas a gender difference was not observed in 7-year-old children. Studies from other countries reported discrepant results regarding the association between gender and abdominal obesity. The prevalence of abdominal obesity was higher in boys in Portugal [38], did not differ between boys and girls in Norway and Spain [16, 27] and was higher in girls in Sweden and in the United States [25, 39]. It has been suggested that longer hours of playing electronic games and watching television might play a role in the higher rates of abdominal obesity in boys [52–55]. Differences in these habits between various countries and age groups might explain the discrepant results regarding the association between gender and abdominal obesity.

The prevalence of abdominal obesity and of obesity defined according to BMI was similar in children living in Crete or other islands and in those living in the mainland. These findings highlight a rapid transition of the population throughout Greece to an unhealthy lifestyle. Indeed, in the pivotal Seven Countries Study performed in the early 60’s, the Cretan population showed the lowest mortality rates from cardiovascular disease [56]. However, subsequent studies showed an increasing prevalence of childhood obesity in Crete [57, 58]. Regarding the association between living in an island and obesity, it is worth mentioning that studies performed in Spain and Portugal also reported that the rates of abdominal obesity and obesity defined according to BMI are similar or higher in children living in islands compared with those living in the mainland [16, 24, 30]. These findings suggest that even though residents of islands might have easier access to more healthy food and more opportunities to exercise, they appear to have adopted a similar lifestyle with children living in the mainland. On the other hand, income and education level are lower in residents of islands in Greece than in residents of mainland [59, 60] and this might also increase the risk of obesity in children living in the islands [61, 62].

We report a higher prevalence of abdominal obesity in both boys and girls living in Athens, the capital of Greece, than in those living in villages and cities with population smaller than 200,000 inhabitants. In contrast, the prevalence of obesity defined according to BMI did not differ between children living in urban or rural areas, again suggesting that abdominal obesity is more sensitive in identifying pediatric populations at risk. Interestingly, in a nationwide study conducted in Greece in 2003, the prevalence of both abdominal obesity and obesity defined according to the BMI did not differ between children living in rural, semi-urban and urban settings [40]. Therefore, it appears that obesity is becoming more prevalent particularly in children living in Athens. Even though the rates of abdominal obesity were lowest in children living in Thessaloniki, the second largest city in Greece, with a population of approximately 1 million, this finding might be misleading since earlier studies reported considerably lower rates of overweight and obesity in children living in this city (25.3 and 5.6%, respectively)[63]. On the other hand, our findings are in contrast with studies from other European countries and from the United States, which reported lower prevalence of BMI-defined obesity and abdominal obesity in children living in cities than in those living in rural areas [23, 24, 26–28]. This discrepancy might be due to differences between rural areas in these countries and Greece in lifestyle and socioeconomic factors [28, 61, 62]. The prevalence of obesity appears to be inversely associated with the socioeconomic status and the abrupt urbanization in Greece might have resulted in worsening living condition in families moving to Athens [61, 62]. Indeed, in a recent study in 4,538 Swedish children 7–9 years old, the prevalence of overweight and obesity was higher in rural areas than in urban areas, but these differences became non-significant after adjusting for area education level [25]. Moreover, older studies from the United States reported a higher prevalence of childhood obesity in metropolitan areas [64].

The present study was conducted in 2010–2011, at the beginning of the economic crisis that occurred in Greece. Thus, the prevalence of overweight and abdominal obesity in Greek children may have since increased due to the economic recession that could lead to increased purchase of low-cost, processed, high-fat food and consequently to poor nutrition and health [65].

Our study has several limitations. First, we did not evaluate the cardiometabolic profile of our population and it is therefore unclear which index of obesity more accurately reflects cardiovascular risk. Another limitation was that we did not record the ethnicity in our study. Non-native adolescents in Greece appear to have lower prevalence of obesity than natives [66]. However, the proportion of non-native children in Greece is rather small (<10%) and is therefore unlikely to substantially affect our findings.

## Conclusions

The prevalence of abdominal obesity in Greece is among the highest worldwide and appears to be increasing. Boys and children living in Athens appear to be at higher risk for becoming obese. Given that abdominal obesity is more prevalent than obesity defined according to BMI and appears to be more sensitive in identifying cardiovascular risk, measurement of waist circumference might have to be incorporated in the screening for childhood obesity. In addition, preventive measures focusing at the promotion of physical activity, healthy eating and nutritional education in children, families and school environments are urgently needed to curb the rising prevalence of obesity in Greece.

## Additional files

Additional file 1: Body mass index (BMI) cut-off values for overweight and obesity for children aged 7.0–7.9 and 9.0–9.9 years according to the World Health Organization (WHO) and the International Obesity Task Force (IOTF) definitions. (DOCX 57 kb)
Additional file 2: Raw data. (XLS 1170 kb)
