# Supplementary material for: Prevalence and geographic variation of abdominal obesity in 7- and 9-year-old children in Greece; World Health Organization Childhood Obesity Surveillance Initiative 2010
Source: BMC Public Health. 2017 Jan 28;17:126. doi: 10.1186/s12889-017-4061-x (PMC5273825; doi:10.1186/s12889-017-4061-x)
Supplement: Additional file 1: — Body mass index (BMI) cut-off values for overweight and obesity for children aged 7.0–7.9 and 9.0–9.9 years according to the World Health Organization (WHO) and the International Obesity Task Force (IOTF) definitions. (DOCX 57 kb) [file 12889_2017_4061_MOESM1_ESM.docx]

**Additional file 1.** Body mass index (BMI) cut-off values for overweight and obesity for children aged 7.0-7.9 and 9.0-9.9 years according to the World Health Organization (WHO) and the International Obesity Task Force (IOTF) definitions.

|  | Overweight | | | | Obesity | | | |
| --- | --- | --- | --- | --- | --- | --- | --- | --- |
| Age | Boys | | Girls | | Boys | | Girls | |
| years : months | WHO^a^ | IOTF^b^ | WHO^a^ | IOTF^b^ | WHO^c^ | IOTF^d^ | WHO^c^ | IOTF^d^ |
| 7:0 | 17.05 | 17.88 | 17.29 | 17.69 | 19.02 | 20.59 | 19.79 | 20.39 |
| 7:1 | 17.08 | 17.91 | 17.32 | 17.73 | 19.07 | 20.66 | 19.85 | 20.47 |
| 7:2 | 17.11 | 17.95 | 17.35 | 17.78 | 19.12 | 20.74 | 19.90 | 20.55 |
| 7:3 | 17.14 | 17.99 | 17.38 | 17.82 | 19.17 | 20.82 | 19.96 | 20.63 |
| 7:4 | 17.17 | 18.04 | 17.42 | 17.87 | 19.22 | 20.90 | 20.02 | 20.72 |
| 7:5 | 17.20 | 18.08 | 17.45 | 17.91 | 19.27 | 20.98 | 20.09 | 20.80 |
| 7:6 | 17.23 | 18.12 | 17.49 | 17.96 | 19.33 | 21.06 | 20.15 | 20.89 |
| 7:7 | 17.26 | 18.17 | 17.53 | 18.01 | 19.38 | 21.14 | 20.21 | 20.98 |
| 7:8 | 17.30 | 18.21 | 17.56 | 18.07 | 19.44 | 21.22 | 20.28 | 21.07 |
| 7:9 | 17.33 | 18.26 | 17.60 | 18.12 | 19.50 | 21.30 | 20.35 | 21.16 |
| 7:10 | 17.37 | 18.31 | 17.65 | 18.17 | 19.56 | 21.39 | 20.42 | 21.25 |
| 7:11 | 17.40 | 18.36 | 17.69 | 18.23 | 19.62 | 21.47 | 20.49 | 21.35 |
| 9:0 | 17.91 | 19.07 | 18.33 | 18.99 | 20.47 | 22.71 | 21.51 | 22.66 |
| 9:1 | 17.95 | 19.13 | 18.38 | 19.05 | 20.54 | 22.82 | 21.60 | 22.77 |
| 9:2 | 18.00 | 19.19 | 18.44 | 19.12 | 20.61 | 22.92 | 21.68 | 22.88 |
| 9:3 | 18.04 | 19.25 | 18.49 | 19.18 | 20.69 | 23.03 | 21.77 | 22.99 |
| 9:4 | 18.09 | 19.31 | 18.55 | 19.24 | 20.76 | 23.13 | 21.86 | 23.09 |
| 9:5 | 18.13 | 19.37 | 18.61 | 19.31 | 20.84 | 23.24 | 21.94 | 23.20 |
| 9:6 | 18.18 | 19.43 | 18.67 | 19.38 | 20.92 | 23.34 | 22.03 | 23.31 |
| 9:7 | 18.23 | 19.49 | 18.73 | 19.44 | 20.99 | 23.45 | 22.12 | 23.42 |
| 9:8 | 18.28 | 19.55 | 18.79 | 19.51 | 21.07 | 23.55 | 22.21 | 23.53 |
| 9:9 | 18.33 | 19.61 | 18.85 | 19.58 | 21.15 | 23.66 | 22.30 | 23.64 |
| 9:10 | 18.38 | 19.67 | 18.91 | 19.64 | 21.23 | 23.76 | 22.39 | 23.75 |
| 9:11 | 18.43 | 19.74 | 18.97 | 19.71 | 21.32 | 23.86 | 22.48 | 23.86 |

^a^WHO cut-off values for overweight are defined to pass through a BMI of 25 kg/m^2^ at the age of 19 years [26]. Overweight is defined as a BMI greater than the given value.

^b^IOTF cut-off values for overweight are defined to pass through a BMI of 25 kg/m^2^ at the age of 18 years [27]. Overweight is defined as a BMI equal to or greater than the given value.

^c^WHO cut-off values for obesity are defined to pass through a BMI of 30 kg/m^2^ at the age of 19 years [26]. Obesity is defined as a BMI greater than the given value.

^d^IOTF cut-off values for obesity are defined to pass through a BMI of 30 kg/m^2^ at the age of 18 years [27]. Obesity is defined as a BMI equal to or greater than the given value.
